# Perceptions Toward the Usefulness and Benefits of Teledentistry in the Ministry of National Guard Health Affairs (MNGHA) in Saudi Arabia

**DOI:** 10.7759/cureus.43792

**Published:** 2023-08-20

**Authors:** Sara Aldhamen, Bakheet Al Dosari

**Affiliations:** 1 Health Informatics, College of Public Health and Health Informatics, King Saud Bin Abdulaziz University for Health Sciences (KSAU-HS), Riyadh, SAU; 2 Health Informatics, King Saud Bin Abdulaziz University for Health Sciences (KSAU-HS), Riyadh, SAU

**Keywords:** saudi arabia, mngha, attitudes, perception, knowledge, dentists, teledentistry

## Abstract

Aim

The present study aimed to evaluate the dentists’ perceptions of the usefulness and benefits of teledentistry at the Ministry of National Guard Health Affairs (MNGHA) in Saudi Arabia.

Materials and methods

A cross-sectional study was carried out among 170 dentists from all the dental specialties who were recruited and trained under MNGHA in Saudi Arabia. A questionnaire was distributed using different social media channels. The questions were arranged into four distinct categories: concerns about data security held by dentists, teledentistry and the enhancement of dental practices, and the usefulness of teledentistry for dental clinics and their patients. The responses of the enrolled participants were further collected. The frequency distribution was calculated. A one-way ANOVA test was applied for comparisons. The confidence interval and p-value were set at 95% and ≤ 0.05, respectively.

Results

Most participants showed neutral responses to the perception of teledentistry’s ability to diagnose accurately. Most of them agreed that the waiting list could be shortened with the help of teledentistry and that it would improve the interaction between peers. The mean score for the usefulness of teledentistry in dental practice was found to be significantly higher among 45-54-year-old than other age groups, among those with >16 years of work experience, among consultants, and among those with video conference as the preferred method of communication.

Conclusion

General knowledge, attitudes, and views of the value and advantages of teledentistry were moderate among dentists in the MNGHA. A few issues about diagnostic precision, cost, and data security were relevant.

## Introduction

Technologies for information and communication are used in teledentistry (TD) for treatment planning and remote dental consultation [1]. TD meetings may be carried out in a variety of ways. The most popular kind of teleconsultation is the synchronous technique, commonly known as “real-time consultation,” in which patients and their dentists employ videoconferencing and other communication technology to consult with specialists in other dental practices. Asynchronous TD, sometimes known as the “store-and-forward method,” is the name of the second form. This comprises exchanging and transferring clinical information, such as images, X-rays, and records of the dentist’s extraoral and intraoral examinations, to specialists for assessment and care planning [2].

The COVID-19 outbreak, declared a global pandemic by the World Health Organization, suddenly put pressure on healthcare systems in many parts of the world. In fact, the risk of infection was higher for dental professionals and workers. Transmission can happen when someone comes close to a sick person and immediately makes physical contact with their saliva, intraoral cavity membrane, or infected instruments or equipment [3]. Water irrigation to cool the dental or surgical site during dental procedures can potentially generate aerosols and droplets. Such droplets may remain in the environment for long periods of time and cause transmission over a 1 m radius [4,5]. The main goal while applying the practice of TD was to minimize person-to-person contact due to the persistence of the COVID-19 pandemic and its potential to become endemic. To continue practicing dentistry during and after COVID-19, TD was noted to be a feasible option [6].

Studies have demonstrated the many advantages of TD, including the potential to expand access to dental services, improve their delivery, and boost their cost-effectiveness [7]. Furthermore, it can lessen disparities in oral healthcare between rural and urban settings [8].

Saudi Arabia is progressing its initiatives into a new era in important sectors like health, economics, and technology thanks to the implementation of a comprehensive reform strategy known as "Vision 2030" [9]. Since it has the potential to significantly impact the accomplishment of the three main goals of Vision 2030-" Improve healthcare service," "Develop the e-government," and " Improve the quality of services provided to citizens"-TD appears to be a crucial research area in Saudi Arabia. Teledentistry was virtually as accurate at diagnosing dental caries as non-telemedicine approaches in a systematic evaluation of the subject [10]. However, the quality of the tools, photos, and training would probably affect the viewpoint and precision of oral diagnosis in teledentistry [11].

The current study aims to assess how Saudi Arabia's Ministry of National Guard Health Affairs (MNGHA) dentists perceive the value and advantages of teledentistry. With a focus on how TD can advance dentistry specialty, the study's goal is to help dentists better comprehend, perceive, and evaluate the value of TD two years after the COVID-19 outbreak. The data's accessibility could help practitioners, healthcare executives, and governments encourage the wider use of TD during and after the crisis. The findings will also strengthen the original validity of the investigations. Furthermore, the original study's conclusions are more likely to have wider implications, and future research will probably cover more ground.

## Materials and methods

Study design and participants

This was survey-based cross-sectional research that included a convenient sample of dentists from all the dental specialties who are presently recruited and trained under MNGHA in Saudi Arabia. Exclusion criteria were non-licensed dentists in Saudi Arabia, dentists who are not working at the MNGHA, undergraduate dental students, dental hygienists and assistants, and respondents not willing to fill out the questionnaire.

Ethical permission for the study was obtained through the King Abdullah International Medical Study Center (KAIMRC) (IRB number IRB/1912/22).

Using the statistical program OpenEpi, the sample size was determined based on 170 population size, a 95% confidence interval, 80% power, and an assumed 50% knowledge level. The estimated sample size consisted of 119 participants. The questionnaire was distributed electronically.

Survey instrument

The survey was taken from previous research that was conducted in a similar way [10]. The previous research study assessed the perspectives of dental practitioners in Australia regarding the benefits of telemedicine and its contributions to enhancing dental practice and patient experience. The initial section of the survey asked about career and demographic questions and the choice of one's preferred way of communicating. The second section of the survey was comprised of 5-point Likert scale questions and contained a total of 26 questions. These questions were arranged into four distinct categories, which were as follows: concerns about data security held by dentists, teledentistry and the enhancement of dental practices, and the usefulness of teledentistry for both dental clinics and their patients.

Data collection

An e-mail list of dentists was gathered from the MNGHA website directory staff section. The survey was prepared through Google Forms and distributed online through e-mails and social media platforms, including LinkedIn and Twitter, from the end of September until the first week of December 2022. A concise explanation of the aim of the questionnaire was provided, along with a definition of teledentistry and its advantages and potential applications in everyday practice. After that, a permission statement (consent) to participate in the survey was presented to the respondent. As a method for following up with the dentists, recall emails and messages were sent once every week to all the dentists on the list.

Statistical analysis

The recorded data was compiled and entered in a spreadsheet computer program (Microsoft Excel 2010) and then exported to the data editor page of IBM Corp. Released 2013. IBM SPSS Statistics for Windows, Version 22.0. Armonk, NY: IBM Corp. Frequency distribution was calculated. One-way ANOVA test was applied for comparisons. Confidence interval and p-value were set at 95% and ≤ 0.05, respectively.

## Results

Table 1 shows that most study participants belonged to the 20-34 age group. There were an equal number of males and females. The majority of them had work experience of up to five years. Most of them were general dental practitioners, followed by consultants. Maximum had their preferred method of communication as in-person.

**Table 1:** Distribution of the study population according to demographic characteristics.

Demographic variables	Number	Percentage
Age group (years)		
20-34	67	59.8
35-44	29	25.9
45-54	14	12.5
55-64	2	1.8
Gender		
Male	56	50
Female	56	50
Work experience (years)		
0-5	50	44.6
6-10	20	17.9
11-15	27	24.1
>16	15	13.4
Qualifications		
Consultant/specialist	40	35.7
General Dental Practitioner	43	38.4
Resident/Graduate research dentist	29	25.9
Preferred method of communication		
Forum	3	2.7
Video conference	1	0.9
Social Media	15	13.4
E mail	28	25
Phone	18	16.1
In person	47	42
Total	112	100

Table 2 shows that most study participants were little concerned and very concerned about data security and patient consent. A very small proportion of participants were not concerned at all.

**Table 2:** Frequency of responses to concerns about data security and patient consent.

Questions	Very concerned n (%)	Little concerned n (%)	Not feeling either way n (%)	Not particularly concerned n (%)	Not concerned at all n (%)
Gaining patient consent for teleconsultation	27 (24.1)	36 (32.1)	22 (19.6)	18 (16.1)	9 (8)
Confidentiality when data are sent online	34 (30.4)	36 (32.1)	17 (15.2)	18 (16.1)	7 (6.3)
Potential for digital forgery	30 (26.8)	39 (34.8)	23 (20.5)	17 (15.2)	3 (2.7)
Incompatible hardware and software	25 (22.3)	39 (34.8)	22 (19.6)	18 (16.1)	8 (7.1)
Reliability of tele dental equipment	26 (23.2)	36 (32.1)	27 (24.1)	15 (13.4)	8 (7.1)

Most participants showed neutral responses to the perception of the capability of teledentistry to provide an accurate diagnosis. Most of them agreed that the waiting list could be shortened with the help of teledentistry and that it would improve the interaction between peers. Also, most participants agreed that it would provide a safe atmosphere for practicing dentistry and make patients referrals more efficient (Table 3).

**Table 3:** Frequency of responses to perceptions about the capability of teledentistry to improve practice.

Questions	Disagree strongly n (%)	Disagree n (%)	Neutral n (%)	Agree n (%)	Agree strongly n (%)
Teledentistry would provide accurate diagnosis in a clinical setting	15 (13.4)	19 (17)	41 (36.6)	27 (24.1)	10 (8.9)
Teledentistry would help shorten the waiting list	4 (3.6)	5 (4.5)	20 (17.9)	53 (47.3)	30 (26.8)
Teledentistry would enhance guidelines and advice	3 (2.7)	3 (2.7)	31 (27.7)	58 (51.8)	17 (15.2)
Teledentistry would improve the interaction between peers	2 (1.8)	7 (6.3)	26 (23.2)	58 (51.8)	19 (17)
Teledentistry would provide a safe atmosphere for practicing dentistry (e.g., COVID-19 pandemic)	5 (4.5)	3 (2.7)	20 (17.9)	53 (47.3)	31 (27.7)
Tele dentistry would make patient’s referral more efficient	2 (1.8)	5 (4.5)	16 (14.3)	60 (53.6)	29 (25.9)

Table 4 showed that the majority of participants agreed that teledentistry would enhance clinical training and continuing education and reduce dental practice costs. Also, most of them agreed that it would increase treatment time spent with the patient, necessitate an extra appointment for taking photographs, save time compared with a referral letter, and provide adequate diagnostic information. However, the majority also agreed on the fact that it would be too expensive to set up.

**Table 4:** Frequency of responses to perceptions about the usefulness of teledentistry for dental practice.

Questions	Disagree strongly n (%)	Disagree n (%)	Neutral n (%)	Agree n (%)	Agree strongly n (%)
Tele dentistry would enhance clinical training and continuing education	5 (4.5)	13 (11.6)	30 (26.8)	51 (45.5)	13 (11.6)
Tele dentistry would reduce costs for the dental practices	3 (2.7)	17 (15.2)	26 (23.2)	53 (47.3)	13 (11.6)
Tele dentistry would increase treatment time spent with the patient	5 (4.5)	21 (18.8)	28 (25)	47 (42)	11 (9.8)
Tele dentistry would necessitate an extra appointment for taking photographs	5 (4.5)	14 (12.5)	27 (24.1)	49 (43.8)	17 (15.2)
Tele dentistry would save time compared with a referral letter	4 (3.6)	6 (5.4)	22 (19.6)	65 (58)	15 (13.4)
Tele dentistry would be too expensive to set up	8 (7.1)	22 (19.6)	32 (28.6)	34 (30.4)	16 (14.3)
Tele dentistry would provide adequate diagnostic information	6 (5.4)	25 (22.3)	34 (30.4)	42 (37.5)	5 (4.5)

Table 5 shows that most participants agreed that teledentistry would save patients money, improve communication with patients, be helpful in patient education, and help avoid unnecessary travel to dental clinics. Also, the majority of participants agreed that teledentistry would be convenient and well received by patients, would be useful for patients in remote areas, and should be covered by dental insurance plans.

**Table 5:** Frequency of responses to perceptions about the usefulness of teledentistry for patients.

Questions	Disagree strongly n (%)	Disagree n (%)	Neutral n (%)	Agree n (%)	Agree strongly n (%)
Tele dentistry would save money for patients	3 (2.7)	6 (5.4)	38 (33.9)	49 (43.8)	16 (14.3)
Tele dentistry would improve communication with patients	1 (0.9)	11 (9.8)	21 (18.8)	66 (58.9)	13 (11.6)
Tele dentistry would be helpful patient education	2 (1.8)	4 (3.6)	21 (18.8)	65 (58)	20 (17.9)
Tele dentistry would help to avoid unnecessary travel to Dental clinic	2 (1.8)	8 (7.1)	9 (8)	58 (51.8)	35 (31.3)
Tele dentistry would be helpful in monitoring the patient's condition	3 (2.7)	8 (7.1)	14 (12.5)	60 (53.6)	27 (24.1)
Tele dentistry would be convenient and well received by patients	3 (2.7)	11 (9.8)	35 (31.3)	47 (42)	16 (14.3)
Tele dentistry would be useful for patients in remote areas	2 (1.8)	4 (3.6)	9 (8)	57 (50.9)	40 (35.7)
Tele dentistry should be covered by dental insurance plans.	4 (3.6)	8 (7.1)	19 (17)	48 (42.9)	33 (29.5)

Dentists perceived the application of teledentistry to be most prevalent in Community Dentistry, followed by Oral Medicine, Dental Hygiene and Oral Radiology. The lowest application was perceived in Pedodontics (Table 6).

**Table 6:** Frequency of responses to perceptions of dentists about the application of teledentistry in branches of dentistry.

Branch of dentistry	Proportion of dentists supporting application of Tele dentistry n (%)
Operative Dentistry	40 (35.7)
Prosthodontics	35 (31.3)
Endodontics	43 (38.4)
Orthodontics	52 (46.4)
Periodontics	41 (36.6)
Pedodontics	28 (25)
Oral Medicine	68 (60.7)
Oral Surgery	34 (30.4)
Oral Radiology	58 (51.8)
Community Dentistry	90 (80.4)
Dental Hygiene	65 (58)

Test applied: One-way ANOVA, Independent t-test, *indicates a statistically significant difference.

The mean score for the usefulness of teledentistry in dental practice was significantly higher among 45-54-year-olds than other age groups, among those with >16 years of work experience, among consultants, and among those with video conference as the preferred method of communication. Also, the mean score for the usefulness of teledentistry for patients was found to be significantly higher among 45-54-year-old than in other age groups, among those with >16 years of work experience, among consultants, and among those with video conference and phone as the preferred method of communication (Table 7).

**Table 7:** Comparative assessment of mean teledentistry domains according to demographic variables.

Demographic variables	Data security and patient consent (Mean ± SD)	Capability of tele dentistry to improve practice (Mean ± SD)	Usefulness of tele dentistry for dental practice (Mean ± SD)	Usefulness of tele dentistry for patients (Mean ± SD)
Age group (years)				
20-34	2.39 ± 0.95	3.66 ± 0.78	3.24 ± 0.69	3.66 ± 0.78
35-44	2.52 ± 0.94	3.79 ± 0.49	3.59 ± 0.56	4.00 ± 0.37
45-54	2.50 ± 1.16	4.21 ± 0.69	4.14 ± 0.66	4.29 ± 0.46
55-64	3.50 ± 0.71	4.00 ± 0.00	4.00 ± 0.00	4.00 ± 0.00
p-value	0.436	0.064T	0.000*	0.006*
Gender				
Male	2.46 ± 0.93	3.68 ± 0.71	3.45 ± 0.71	3.82 ± 0.76
Female	2.45 ± 1.02	3.86 ± 0.72	3.46 ± 0.73	3.84 ± 0.62
p-value	0.923	0.192	0.896	0.893
Work experience (years)				
0-5	2.30 ± 0.81	3.72 ± 0.75	3.24 ± 0.68	3.62 ± 0.75
6-10	2.50 ± 1.10	3.30 ± 0.80	3.20 ± 0.69	3.70 ± 0.80
11-15	2.74 ± 0.98	4.04 ± 0.43	3.81 ± 0.68	4.11 ± 0.42
>16	2.40 ± 1.24	4.07 ± 0.59	3.87 ± 0.51	4.20 ± 0.41
p-value	0.303	0.001*	0.000*	0.002*
Qualifications				
Consultant/specialist	2.48 ± 1.03	3.83 ± 0.78	3.63 ± 0.77	4.03 ± 0.66
General Dental Practitioner	2.26 ± 1.00	3.67 ± 0.81	3.21 ± 0.67	3.56 ± 0.79
Resident/Graduate research dentist	2.72 ± 0.79	3.83 ± 0.46	3.59 ± 0.62	3.97 ± 0.42
p-value	0.135	0.562	0.016*	0.004*
Preferred method of communication				
Forum	1.67 ± 1.15	3.67 ± 0.57	3.33 ± 0.57	3.33 ± 0.57
Video conference	4.00 ± 0.00	5.00 ± 0.00	4.00 ± 0.00	4.00 ± 0.00
Social Media	2.47 ± 0.91	3.20 ± 0.94	3.07 ± 0.70	3.27 ± 0.70
E mail	2.29 ± 1.01	3.82 ± 0.81	3.21 ± 0.95	3.82 ± 1.02
Phone	2.72 ± 1.01	3.83 ± 0.51	3.50 ± 0.51	4.00 ± 0.48
In person	2.47 ± 0.92	3.87 ± 0.57	3.70 ± 0.55	3.98 ± 0.39
p-value	0.248	0.016*	0.015*	0.01*

## Discussion

The current study aims to survey and evaluate the values and benefits of teledentistry as perceived by the doctors of Saudi Arabia's Ministry of National Guard Health Affairs (MNGHA). The survey and report will aid dentists in better comprehending the utility of TD in the face of an epidemic and incentivize its more widespread use. The term "telehealth" describes the delivery of medical services and care utilizing electronic and technological networks [12]. Over the past decade, information and communication technology has gained popularity in healthcare, and wireless connectivity has become more widespread as tech devices like smartphones and applications have become more commonly available and affordable [13]. Teledentistry (TD) is the discipline of dentistry that delivers care, teaching, research, and supervision from a distance through technology-enabled communication and information sharing [14]. Dental specialists worldwide are gradually embracing it because it is a relatively new area. Developing technologies have already altered and transformed how oral health care services are given to patients [15].

The majority of study participants belonged to the 20-34 age group [13], and most of them were General dental practitioners, followed by consultants. Most of the enrolled participants favored in-person communication. The majority of study participants were little concerned and very concerned about data security, patient consent, and patients' personal information being shared online. Due to the increased use of technology in practice, there is concern about the security risks associated with unencrypted data transfers online. As breaches of patient privacy appear easier than ever before in an electronic environment, maintaining their privacy becomes difficult. To protect patient information shared electronically, teledentistry requires a security framework [16,17]. These findings were consistent with research that found comparable results regarding worries about data privacy [18].

Most participants showed neutral responses to the perception of teledentistry's capability to provide an accurate diagnosis. Most of them agreed that the waiting list can be shortened with the help of teledentistry and would improve the interaction between peers. Also, most participants agreed it would provide a safe atmosphere for practicing dentistry and make patient referrals more efficient. Compared to the clinical setting, there was a lot of doubt about whether teledentistry could aid in accurate diagnosis, as reported in many studies [10,19]. Other investigations reported positive diagnostic accuracy when identifying an oral disease or caries [10]. This was accomplished after thorough instruction, where practicing dentists led training sessions for accurate diagnosis. Thus, teledentistry can produce 15 acceptable diagnoses with the right training and adherence to a predetermined protocol. The fact that more than half of respondents said taking additional photos for teledentistry would be necessary demonstrates their awareness of the importance of having professional photos taken [10].

Teledentistry could, from the standpoint of medical practice, reduce the waiting period and duration between consultations [16]. The online connection procedure between clinics or dentists is quicker than the conventional paper referral, which takes more time, as patients' conditions and demands are exchanged sooner, and patients are referred to a dental specialist clinic predicated on an initial informed decision and necessity [15]. Additionally, teledentistry enables multi-participant conferences to debate a case; hence, engagement between medical professionals enhances the standard of care that benefits patients. As a result, a conversation between a specialist and a general doctor who is referring patients helps with clinical training and ongoing education [20].

The majority of participants agreed that teledentistry would enhance clinical training and continuing education and reduce dental practice costs. Also, most of them agreed that it would [17]provide additional clinical time for procedures for the patients. Most of the dentists in this study agreed on the fact that it would be too expensive to set up. One reason for this might be that TD requires a stable network as well as technological and security support to get started, yet there is very little published evidence regarding the estimated set-up costs for teledentistry. According to a study that was conducted to explore the challenges and mitigation strategies towards practicing TD, the set up costs were one of the challenges for establishing TD in a private dental office because practitioners may have to pay for it themselves, with few financial benefits [21]. However, our study results contrasted among dental professionals in Pakistan and Saudi Arabia in a survey, which reported that the least number of participants agreed that TD would be too expensive to set up [22].

Most participants agreed that teledentistry would save patients money, improve communication with patients, be helpful in patient education, and help avoid unnecessary travel to the dental clinic. Also, most participants agreed that teledentistry would be convenient and well-received by patients. It would be useful for patients in remote areas, and dental insurance plans should cover it. The majority of participants in a reported research survey agree that it was more cost-effective for the patients; as sessions could be conducted online, superfluous travel is avoided, which is useful in particular for patients in rural places [22]. Only 21% of dental practitioners in India consider that teledentistry reduces the costs of dental practices, despite reports that the cost of patient examinations using this technology is less than that of traditional means [23]. As a result, it saves time because less time is spent getting there, awaiting the scheduled consultation, and hanging out at the clinic. Only 33.3% of respondents in India believe that teledentistry saves time, even though other research has demonstrated that effective time management and cost-saving are important in winning patients' confidence and motivating them to adopt teledentistry [24]. Reports suggest the fact that there are three main ways it can be applied as a service paradigm, such as conversations between dentists to exchange patient photographs and documents, accompanied by a review and therapeutic intervention planning discussion; a face-to-face video conference discussion instantaneously between a general dentist and a patient in a far-off, remote place; and remote patient surveillance, which collects data in real-time and sends it to the dentist in a far-off location for illustration [25].

Dentists perceived the application of teledentistry to be most prevalent in Community Dentistry, followed by Oral Medicine, Dental Hygiene and Oral Radiology. This result is comparable to those obtained in a previous study conducted in Saudi Arabia and Pakistan [22]. The lowest application was perceived in pedodontics. Compared to Pakistani dentists, a greater percentage of participants from Saudi Arabia were employed in the public sector and had fewer hours of labor per week but were noted to be in favor of teledentistry [22]. Government services may also be affected by teledentistry. Hence, efforts to increase and expedite the adoption rate of teledentistry should be supported.

The mean score for the usefulness of teledentistry in dental practice was significantly higher among 45-54-year-old than other age groups, among those with >16 years of work experience, among consultants, and among those with video conference as the preferred method of communication. The qualifications and professional experience of the participants had a big impact on the findings of this study [20]. It was reported that general dentists performed worse than the other groups, which may be likely because they were less accustomed to using new technology or could not comprehend it [26]. Other research has demonstrated the appeal and advantages of tele-dental applications for general dentists, who might learn from and occasionally carry out more complex treatments under tele-supervision [6,27]. The middle-aged group of dentists, who had worked for 11 to 15 years, were noted to have the lowest score as they may be still figuring out how to establish their careers and may be averse to the technology so as not to compromise their patient base [26].

To properly use teledentistry in dental practice, more research is required. This infrastructure may include networking, hardware elements, intraoral cameras, and digital radiographs due to the lack of knowledge regarding the level of TD equipment available in Saudi dental clinics and institutes. A qualitative study may be conducted in the future that focuses on the issue of readiness, current restrictions, and potential solutions for improving the integration of TD into Saudi Arabia's oral healthcare system. To ensure the anonymity and privacy of patients while delivering top-notch dental and oral care, it is also crucial to adopt laws and regulations expressly for teledentistry.

The study's restriction to MNGHA dentists was one of its shortcomings. Using a 5-point Likert scale may also have disadvantages because respondents prefer to express their opinions in the middle of the spectrum rather than at the extremes.

## Conclusions

In teledentistry, dental information can be delivered and shared more efficiently, easily, quickly, and safely; thus, it can improve dental services. Adopting teledentistry as an additional or alternative delivery method improves access to healthcare services and reduces the need to travel and the cost of treatment. It benefits populations living in areas with poor infrastructure, a shortage of dentists and dental specialists, and limited oral care services, particularly in developing and underdeveloped countries.

General knowledge, attitudes, and views of the value and advantages of teledentistry were moderate among dentists in the MNGHA. A few issues about diagnostic precision, cost, and data security were relevant. These problems suggest that legislators should carefully specify cost estimation and usage guidelines before teledentistry is widely adopted.
